# What makes inequality in the area of dental and oral health in developing countries? A scoping review

**DOI:** 10.1186/s12962-021-00309-0

**Published:** 2021-08-26

**Authors:** Peivand Bastani, Mohammadtaghi Mohammadpour, Gholamhossein Mehraliain, Sajad Delavari, Sisira Edirippulige

**Affiliations:** 1 Health Human Resources Research Centre, School of Health Management and Medical Informatics, Shiraz University of Medical Sciences, Shiraz, Iran; 2 Student Research Committee, Shiraz University of Medical Sciences, Shiraz, Iran; 3 School of Pharmacy, Shahid Beheshti University of Medical Sciences, Tehran, Iran; 4 Center for Health Services Research, Faculty of Medicine, The University of Queensland, Brisbane, Australia

**Keywords:** Inequality, Oral health, Dental health, Developing countries

## Abstract

**Background:**

Equity in health is an important consideration for policy makers particularly in low and middle income developing country. The area of oral and dental health is not an exception. This study is conducted to explore the main determinants that make inequality in oral and dental health area in developing countries.

**Methods:**

This was a scoping review applying the framework enhanced by Levac et al. Four databases of Scopus, PubMed, WOS and ProQuest were systematically searched applying to related keywords up to 27.11.2020. There restriction was placed in the English language but not on the study design. All the related studies conducted in the low or middle income developing countries were included. A qualitative thematic analysis was applied for data analysis and a thematic map was presented.

**Results:**

Among 436 articles after excluding duplications, 73 articles were included that the number of publications from Brazil was greater than other developing countries (33.33%). Thematic analysis of the evidence has led to 11 determinants that may result in inequality in oral and dental health services in developing countries including personal characteristics, health status, health needs and health behaviours, social, economic, cultural and environmental factors, as well as insurance, policies and practices and provided related factors.

**Conclusion:**

The policymakers in the low and middle income developing countries should be both aware of the role of inequality determinants and also try to shift the resources to the policies and practises that can improve the condition of population access to oral and dental services the same as comprehensive insurance packages, national surveillance system and fair distribution of dentistry facilities. It is also important to improve the population’s health literacy and health behaviour through social media and other suitable mechanisms according to the countries’ local contexts.

**Supplementary Information:**

The online version contains supplementary material available at 10.1186/s12962-021-00309-0.

## Background

The populations’ oral and dental health is among the public health concerns globally. Evidence shows that the distribution and severity of the diseases related to oral and dental health can vary around the world [1]. While some evidence emphasizes that the prevalence of dental caries is decreased among both developing and developed countries [2], other studies show the high prevalence of dental diseases among those populations with low socioeconomic status [3]. Such these contradictions can simply indicate that the issue of oral and dental health needs to be considered yet.

On the other hand, other evidence, indicates that the treatments applied for oral and dental diseases are considered as the 4th expenditures even among industrial and developed countries [4]. Because of the intensive costs and due to the relationship among the frequency of these diseases and the social, behavioural and environmental factors [1], it seems that this issue needs more consideration among low and middle income developing countries. In this regard, the previous studies have shown the inequalities in the area of oral and dental health. Such an inequality can be either due to the unfair provision of the services or each of the inappropriate access or utilization of the services by the population [5]. According to the evidences almost 4.6% of healthcare expenditures globally is allocated to the dental caries and the related treatments. Such an expenditure is varied from one country to the others and in many regions is funded by out of pocket payments at the time of patients’ needs [6] that can intensify the inequality and access to the dental services.

Equity as one of the main aims for the healthcare policymakers is directly pointed to any potential differences in the population’s health including either their financing, access to the services or the health level of the community [7]. According to the evidences, different determinants can lead to inequality in oral and dental diseases. Among them the social, cultural, ethnical, psychological and behavioral factors can be considered [8]. In this regard evidence shows that socio-determinants of health (SDH) the same as education, income, environmental condition, the community’s working life as well of the other factors the same as adequate oral health professionals can play an important role in decreasing disparities and as a results, promoting the health equity [9].

At the same time, the present knowledge indicates that the population’s income along with the cost of dentistry services are among other important determinants that can affect the affordability of the services and consequently intensify the inequality [10]. So, a clear identification of these determinants should be mentioned comprehensively to shed the light for policymakers for better allocation of the resources and equitable provision of oral and dental health services particularly in developing countries.

According to what was said, although the indications of inequality in dental services has been reported in many communities, the challenge is much more highlighted among low- and middle-income countries. According to the evidences, many inconsistency and knowledge gaps are obvious in the area of oral policies among these countries [11, 12] that make the national, local and regional policy makers pay more attention to this area. In another words, to the best of our knowledge, although many contents are considered a single or multiple cause of inequality in the area of oral and dental health, a scoping review in the context of low and middle income developing countries is not presented. Moreover, as the issue of inequality in health is related to the context and setting, the determinant factors may differ from the developed or in transition countries to the developing or under developed ones. Considering all the above, this scoping study is conducted to explore the main determinants that make inequality in oral and dental health area among developing countries. This approach can make an opportunity to consider the whole related scope, and explore all the determinants stated in the related literature to pave the way for health policymakers in developing countries in order to plan based on the evidence and applied to the context.

## Methods

The present scoping review was conducted in November 2020. This kind of reviews, is generally applied to define and clarify the determinants and key concepts of a research scope and map the evidences and conceptual boundaries of the topic [13]. Different frameworks are proposed to conduct a scoping review. First of all, was suggested by Arksey and O’Malley with a five obligatory and an optional consequential steps [13]. This framework has renewed by Levac, Colquhoun and O’Brien [14]. According to Levac et al. all the six steps of the Arksey and O’Malley’s framework was enhanced. In this study the later framework is applied because of more explicit details, clarity and rigor through the review process [15].

### Clarifying and linking the purpose and research question

At the first step of the scoping review the purpose of the study was confirmed as “determination of the main and sub factor affecting inequality in oral and dental health services among developing countries”. According to this purpose the following research question was defined: “What are the main determinants of inequality in access to oral and dental health services”.

### Balancing feasibility with breadth and comprehensiveness of the scoping process

At the second step, the area and scope of seeking the evidences were identified. In this regard, four main databases including PubMed, ISI Web of Science, Scopus and ProQuest were systematically searched. Related keywords were chosen and they were combined applying logical operators OR/AND in order to increase the sensitivity of the search. The main keywords were “dental health”, “oral health”, “socioeconomic”, “healthcare disparities”, “utilization” and “inequality”. Although the aim of the scoping review was to explore the determinants of inequality in oral and dental health among developing countries, “developing country” was not applied as the main key word because many of the studies directly pointed to the name of the developing country not the general term. The search strategy was conducted up to 27 Nov 2020 considering two limitations for time and language. The time limitation was considered from 1 Jan 2000 to 27 Nov 2020 and the language limitations was defined for those articles which has published in a full text format in English. The syntax search is presented in Table 1 according to each of the aforementioned databases. Also, at the end of the process of systematic search, a google search was implemented for retrieving the related pre-prints and unpublished or grey literature in this area.

**Table 1:** The search strategy of the scoping review

Databases	Key words combination
PubMed	((("Dental Health Surveys"[Mesh]) OR ( "Oral Health"[Mesh] OR "Dental Health Services"[Mesh] )) AND ((((("Socioeconomic Factors"[Mesh]) OR "Hierarchy, Social"[Mesh]) OR ( "Healthcare Disparities"[Mesh] OR "Health Status Disparities"[Mesh] )) OR "Social Determinants of Health"[Mesh]) OR "Social Class"[Mesh])) AND (((("dental services"[Title/Abstract]) OR ("dental visits"[Title/Abstract])) OR (utilization[Title/Abstract])) OR ("use of services"[Title/Abstract]))
SCOPUS	TITLE-ABS-KEY("oral health") OR TITLE-ABS-KEY("Dental Health Surveys") OR TITLE-ABS-KEY("Dental Health") OR TITLE-ABS-KEY("dental care") AND TITLE-ABS-KEY("Socioeconomic Factors") OR TITLE-ABS-KEY("Social Hierarchy") OR TITLE-ABS-KEY(Inequalities) OR TITLE-ABS-KEY("Social Disparities") OR TITLE-ABS-KEY("Social Gradient") OR TITLE-ABS-KEY("Health Status") OR TITLE-ABS-KEY("socioeconomic disadvantage") OR TITLE-ABS-KEY("socioeconomic inequalities") OR TITLE-ABS-KEY("Social Determinants") AND TITLE-ABS-KEY("dental services") OR TITLE-ABS-KEY("dental visits") OR TITLE-ABS-KEY("utilization") OR TITLE-ABS-KEY(access) OR TITLE-ABS-KEY("use of services")
WOS	TOPIC: (“Dental Health Surveys” OR “Oral Health Disparities” OR “Dental Health” OR “Oral Health” OR "dental care") (“Socioeconomic Factors” OR “Social Hierarchy” OR “Inequalities” OR “Social Disparities” OR “Social Gradient*” OR “Health Status*” OR “socioeconomic disadvantage” OR “socioeconomic inequalities” OR “Social Determinants” OR “Socio Economic Status”) TOPIC: (“dental services)
ProQuest	(MESH.EXACT("Dental Care") OR MJMESH.EXACT("Dental Health Surveys") OR MJMESH.EXACT("Dental Health Services") OR MJMESH.EXACT("Oral Health")) AND (MJMESH.EXACT("Socioeconomic Factors") OR MESH.EXACT("Social Class") OR MJMESH.EXACT("Social Determinants of Health"))

### Using an iterative team approach to selecting studies and extracting data

Applying the aforementioned search strategy (Table 1), all the four databases were systematically searched. 6521 cases were reached following this strategy. After reviewing the titles, 4535 cases were remained and after screening and omitting the duplications, a total of 436 articles were included. These articles were screened first by their abstracts and the relevant abstracts were completely reviewed by their full texts. In this step, the eligibility of the articles was defined so that, those articles with no English full-texts and those articles with no full-texts format the same as conference proceedings were excluded. Furthermore, those articles in any formats of editorials, commentaries and letters were excluded and were not eligible to analyse because they do not contain any data-driven results. Another screening stage in this step was selecting those studies according to the list of the developing countries based on the World Economic Situation Prospects released by the United Nations 2020 (https://www.un.org/development/desa/dpad/wp-content/uploads/sites/45/WESP2020_Annex.pdf).

In this regard, all the original or review articles with any qualitative or quantitative design derived from any of the developing countries based on the aforementioned list which indicate the aim of the present scoping review were included. Meanwhile, none of the records identified through other sources were eligible for including data analysis step.

**Fig. 1:**
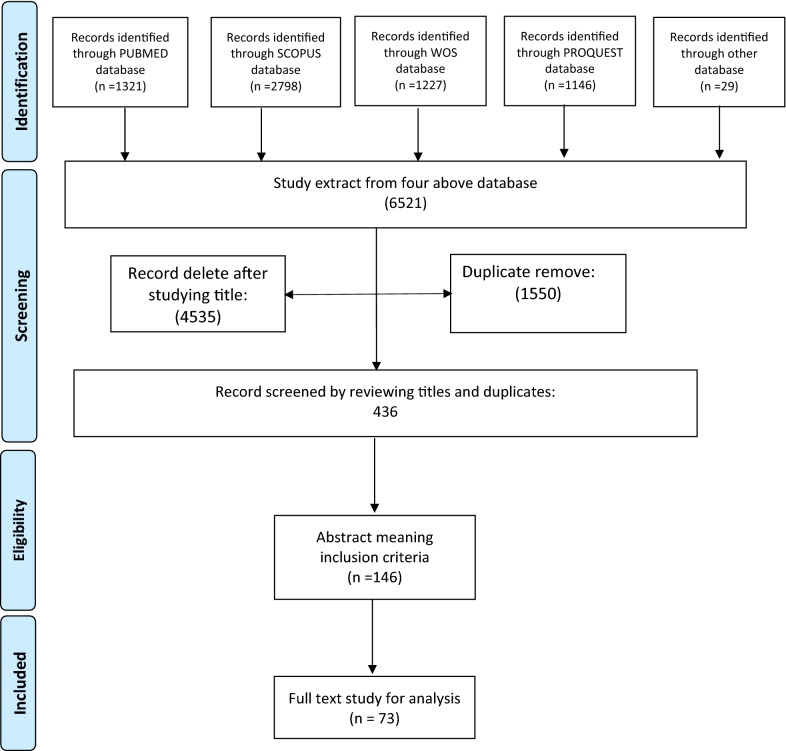
The PRISMA flowchart of the scoping review

For managing the pre-stated process, Endnote X7.1, by Thomson Reuters was applied. Figure 1 shows the PRISMA flowchart.

### Incorporating a numerical summary and qualitative thematic analysis

**Fig. 2:**
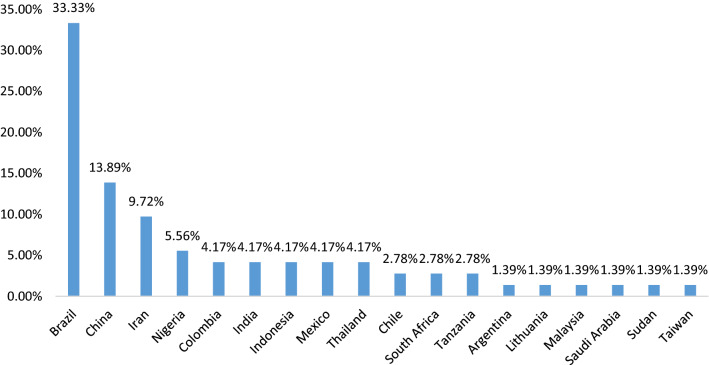
The included articles distribution according to the publication place

In order to extract the data from the included articles, a data extraction form was prepared including the first authors’ name, the year and place of publication, the study aim and design and the main results (Additional file 1: Table S1). Microsoft Excel software version 2013 was applied to extract the data. This step is carefully done by one of the researchers (MM) and the extracted results were described according to the frequency of publications via Fig. 2. For evidence synthesis a qualitative thematic analysis was conducted. For this propose, after extracting the effective factors of oral and dental health inequality from each article as the final code, the new concepts were made by categorizing the codes, the topic charting process was applied via a table to determine which codes belonged to each category.

### Identifying the implications of the study findings for policy, practice or research

A qualitative thematic analysis was conducted to achieve the main and the sub determinants of the inequality in the scope of oral and dental health services as well as the implications for policy makers and oral and dental health providers. For a qualitative thematic analysis [16], first we have familiarized with the data through a continuous process of reviewing the extracted data and comparing it with the included articles, then, the coding process was started and the initial codes were made and ladled through an open coding process based on the research question. Continuing the coding process, the initial codes were refined to reach to the final codes. All the final codes that indicate on any sort of inequality in utilizing the oral and dental services in the mentioned countries were analysed thematically. In a way that, after finalizing the codes, the final emerged codes were categorized and classified to make the sub-themes and then the main themes with more synthesis in a higher conceptuality level. These sub-themes and main themes, then were reviewed and finalized and finally the appropriate labels were chosen and the suitable definition and demonstration of the main and sub themes were presented in a table (Table 2). The qualitative software MAX QDA version 10 was used to analyse the data.

**Table 2:** The main determinants of inequality to oral and dental health access among developing countries

Main themes	Sub-themes	Final codes	References
Micro Individual level	Personal characteristics	Age	[4, 17–19]
		Sex	[20]
		Skin colour	[20, 21]
		Higher self-esteem	[22]
		Gender/child gender	[23]
	Health status	Periodontal status	[24]
		Severity of dental caries	[25]
		Self-rated oral health	[22, 26, 27]
		Systemic disease history	[17]
		Decayed teeth	[28]
		Psychological health status	[29–31]
	Health needs	Dental treatment needs	[28, 32]
		Perceived dental treatment needs	[4, 20, 33, 34]
		Perceived oral health care need	[35]
		Evaluated need characteristics (oral clinical status)	[23]
	Health behaviours	Oral health beliefs	[22]
		Regular brushing	[22, 27]
		Oral hygiene practice	[4]
		Children’s dental behaviours	[36]
		Oral health education for parents and children	[36]
		Oral health knowledge	[36]
Macro level	Social determinants	Rural–urban disparity	[25]
		Unemployment	[25, 37, 38]
		Employment status	[39]
		Need and predisposing factors	[40]
		Education level (mother, household’s head)/ parents’ schooling	[26, 41] [17–19, 32, 34, 37, 42–46]
		Work conditions of the mother	[47]
		Social class/social position of the family head	[8, 47–49]
		Socioeconomic condition	[23, 41, 46, 50, 51]
		Living in rural areas	[38]
		Residential location	[34]
		Urban–rural disparity	[52]
		Educational inequalities	[53, 54]
		Geographical and financial access	[55]
	Economic-determinants	Being poor/poverty	[20, 25, 28]
		prepayment for health services	[55, 56]
		Income	[19, 22, 32, 41, 42, 54–60]
		Financial autonomy	[47, 4]
	Cultural determinants	Cultural values	[47]
		Individual and contextual determinants	[61]
	Environmental determinants	Supporting environment	[49]
		Geographic barriers to dental care	[62]
Mezzo organizational level	Provider related factors	Ratio of dentists to inhabitants	[63]
		Institutions, staff, and providers	[47]
		Absence of a national surveillance system for oral health	[64]
		The fragmentation of actors and institutions	[64]
		Absence of leaders uniting various actors in oral public health	[64]
		Regionally equitable distribution of dentists	[62]
		Caregivers’ oral health knowledge	[17]
		Enhanced provision of oral health care services	[65]
	Policies and practices	Multi-sectoral approach	[65]
		Multi-sectoral collaboration	[65]
		Dental care market competition	[66]
		Institutions, staff, and providers	[47]
		Prioritization of population groups	[47]
		Coverage of the family health strategy	[67]
	Insurance	Supplementary insurance	[67]
		Basic Care Package indicators	[63]
		Type of health insurance	[46, 62, 68]
		Dental health insurance	[27, 54, 60, 69]

### Adopting consultation as a required component of scoping study methodology

In order to achieve an appropriate schematic and understandable map for the policymakers, a thematic map was presented. A mini expert panel was conducted including the research team with sufficient reflexivity in the qualitative studies and thematic analysis and three representatives of national oral and dental health policymaking to finalize the thematic map.

## Results

Results showed that 6521 cases were reached following the present strategy. After reviewing the titles, 4535 cases were remained and after screening and omitting the duplications, a total of 436 articles were included**.** Among 436 articles after excluding duplications, 73 articles were included and extracted.

Descriptive analysis of the included studies showed that most of these articles (33.33%) were published about Brazilian setting. China and Iran have the second and the third proportion of the articles respectively. Figure 2 compares the distribution of the included articles according to the place of publication.

**Fig. 3:**
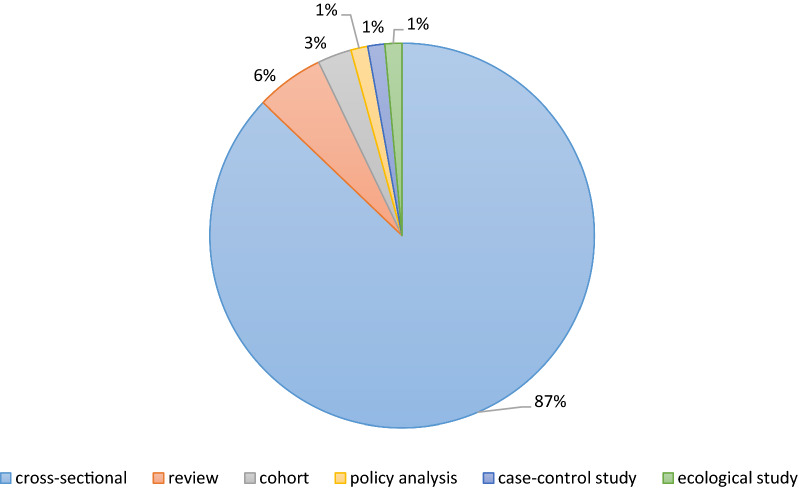
The included articles distribution according to study design

Other results demonstrated that most of the articles (87%) had a cross-sectional design while the policy analysis (1%), ecological studies (1%) and the studies with the case–control design (1%) were among the least methodological approaches. (Fig. 3).

**Fig. 4:**
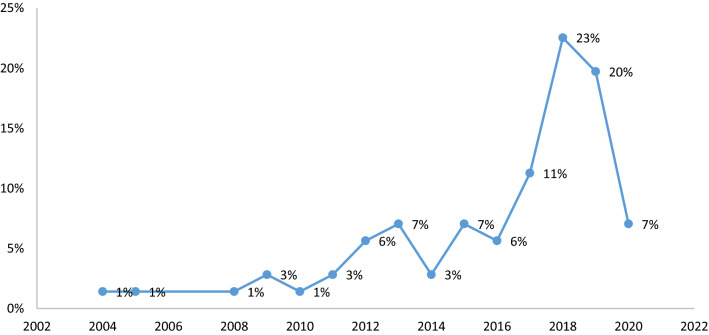
The included articles distribution according to year of publication

Other descriptive results of the study are shown in Fig. 4. According to Fig. 4, there was a rising in the attention to the topic from 2004 to 2018 and most of the articles have been published in 2018.

Thematic analysis of the evidences has led to 11 determinants that may result in inequality in oral and dental health services in developing countries including personal characteristics, health status, health needs and health behaviours, social, economic, cultural and environmental factors, as well as insurance, policies and practices and provided related factors (Table 2).

As Table 2 shows, the aforementioned determinants can affect the equality or inequality of oral and dental health services in three levels.

The first level is the micro-individual level. It is the most related area to the populations attributes and include: personal characteristics of the population, health status of the population and the population’s health needs and health behaviours. In another words, according to the included and analysed literature, some personal characteristics the same as age [18, 19], gender [23] and race [20, 21] can directly and indirectly affect the access to oral and dental health services. These characteristics along with the populations’ physical [17], dental [22, 25–27] and psychological [29–31] health status can determine the health needs and the health behaviours consequently.

The second affecting area on the equality or inequality of oral and dental health is related to the mezzo level that is about the health organizations. One of the main determinants in the mezzo level, according to the included literature was insurance [63, 67]. This factor can determine the health seeking behaviours of the population [60, 69]. More than a coverage for oral and dental health, the providers can have a large effect on the populations’ access to oral and dental health. An equitable distribution of the providers can lead to a larger geographic access and help the oral and dental health equity among the population [62], at the same time, adequate oral and dental health centres and dentistry clinics accompanied with the sufficient and educated staff, existing a surveillance system and an integration among the organizations and actors [64], are among the organizational issues that should be considered in the mezzo-level. It would be obvious that the policies and the practises can both influence on the insurance organizations and the oral and dental services’ providers [65, 66].

**Fig. 5:**
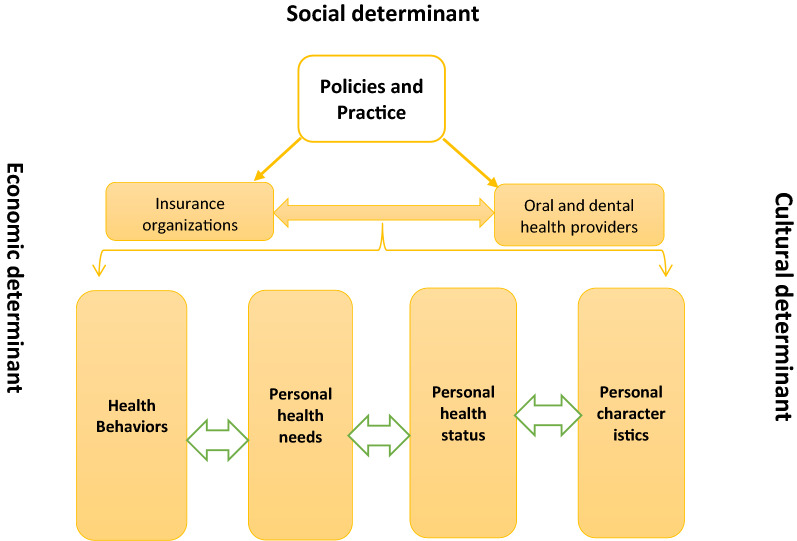
A thematic map of the inequality in oral and dental health services

And finally the third category is related to macro-level factors. Among them we can refer to social, cultural, economic and environmental determinants. These determinants are more related to the social determinants of health (SDH) and can both influence on the populations’ individual conditions and the organizations’ practices and policies. In another words, the social and cultural factors can highly affect the community’s oral and dental health beliefs [22], their oral and dental perceived needs [33, 35] and the population’ level of education and health literacy [36]. The economic determinants, similarly can change the community’s oral and dental health behaviours the same as seeking for consultations, treatments or check-up [55, 56]. More than what was said, the social, cultural, economic and environmental context of a developing country can affect the mezzo-level factors the same as insurance benefit packages, the providers’ practices and the whole policies. Figure 5 has illustrated the relationships in these three levels.

## Discussion

Inequality is a significant concept for health policy makers and the area of oral and dental health can be faced with inequality due to various reasons. Results of this study is comprehensively present the main determinants that can lead to inequality in the area of oral and dental health services.

According to the present results, some personal characteristics can affect inequality in oral and dental health area among developing countries including age, sex, skin color, gender and the influence the population’s health status and lead to emerging different health needs and health behaviors. Rebelo et al. have emphasized that some of the demographic characteristics the same as age, can have a relationship with socioeconomic determinants. For instance, age can have a reverse association with education and health literacy but the positive association with the income [70]. Or elsewhere, Honkala et al. have confirmed a significant correlation among some individual determinants including occupation and education of the population and their dental visits’ frequency [71]. According to these evidences and the present thematic map, a mutual relationship between personal characteristics of the populations and their oral and dental health status can determine their health needs as well as their unmet needs. The later should be particularly considered by the policy makers in low and middle income developing countries. Finally, the present results have demonstrated the mutual relationship of the oral and dental health needs on the population’s behaviour the same as their oral health beliefs, habits, practices and behaviours. In this regard, Thomson has also declared that many oral and dental health behaviours, life style and health experiences are closely related to the social context and structure that can itself be considered as a determinant of health equity or inequality [72].

More than the above determinants that are presented in a micro level, other results of the present study, have confirmed on social, cultural, economic and environmental determinants at the macro level as the main affecting factors that can lead to oral and dental health inequality among the populations in developing countries. Many studies have considered the relation between socioeconomic determinants and health equality, among them we can consider Rezaei et al. that clearly confirmed that a prorich type of inequality is existing in the north of Iran as a developing country and the variable of income is determined as the main cause of such a pro-rich inequality [27]. Another study by Mejia et al. has also shown that there are differences among the level of oral and dental health according to the populations’ income and education and their socioeconomic condition. The authors have also emphasized on the significant inequality in the area of oral and dental health among four industrial developed understudied countries that need the serious health policy interventions on the macro social, cultural and contextual determinants [73].

The present studies have also explored the organizational determinants in the mezzo level. The insurance organizations and the oral and dental health providers. These two organizations have affected by the countries health policies and practices. At the same time the macro level cultural, economic, environmental and social determinants can affect the national and local policies. These macro determinants can play as the macro trends and shift the directions of the policymakers toward facilitating the use of dentistry services for the population, better access and also appropriate provision of these services.

So according to the present thematic map it is obvious that macro determinants consisting of social, cultural, environmental and economic factors can both affect the whole national policies and guide the health policy makers in defining the new agendas or proposing the interventions in order to support the oral and dental health status and decrease the inequalities. Whereas, these macro level determinants can affect the micro level factors the same as the family’s perceived needs related to oral and dental health as well as their unmet needs and the income allocated to respond to these kind of health needs. This thematic map can shed the light for policymakers to better understanding of the determinant factors and their relationships and try to design applied interventions to decrease the inequality in oral and dental area.

More than implications for policymakers, this study can highlight some new areas for future researches as follows: assessment of the impacts of each determinant in disparity and equality of the developing countries, testing the thematic map in a quantitative approach for evaluating the proportion of each factors impact.

## Conclusion

According to the results the policymakers in the low and middle income developing countries should be first aware of the role of determinants that can lead to inequality and then try to shift the resources to the policies and practises that can improve the condition of population access to oral and dental services the same as comprehensive insurance packages, national surveillance system and fair distribution of dentistry facilities. It is also considerable to improve the population’s health literacy and health behaviour through social media and other suitable mechanisms according to the countries’ local contexts.

## Limitations

Two kinds of limitations were encountered in the present study: the first limitation was related to the nature of generalization and applicability of the results. In another words, the inequality determinants may have differently weighted among the context of low and middle income developing and even underdeveloped countries. This can be considered as the first limitation and need to be considered before applying by policymakers in developing countries. At the same time, the second limitation was related to the process of scoping review. In this regard it should be mentioned that the scoping review is restricted to the articles and published materials via four main scientific databases and other sources the same as dental and oral health related databases and websites were not included and analysed.

## Supplementary Information

**Additional file 1: Table S1. **The summary of the included studies characteristics.

## Data Availability

While identifying/confidential patient data should not be published within the manuscript, the datasets used and/or analysed during the current study are available from the corresponding author on reasonable request.

## Abbreviation

MAX QDA: Software for qualitative document analysis
