# Supplementary material for: What makes inequality in the area of dental and oral health in developing countries? A scoping review
Source: Cost Eff Resour Alloc. 2021 Aug 26;19:54. doi: 10.1186/s12962-021-00309-0 (PMC8394054; doi:10.1186/s12962-021-00309-0)
Supplement: Supplementary file 1 — Additional file 1: Table S1. The summary of the included studies characteristics. [file 12962_2021_309_MOESM1_ESM.docx]

**Additional file 1:** Table S1. The summary of the included studies characteristics

|  | First Author (Year) | Country | Aim of the Study | Study Design | Setting |
| --- | --- | --- | --- | --- | --- |
| 1 | Cheng, M. L. | China | Investigate the dental expenditure, analyze its progressivity and horizontal inequality under the general health finance and insurance system, and identify the key social determinants of the inequality for Chinese adults | A secondary analysis | data of 13,464 adults from the 4th National Oral Health Epidemiological Survey (NOHES) in China |
| 2 | Al Agili, D. E. | Saudi Arabia | predict factors that influence utilization of oral health services for children in Saudi Arabia | using the access to care questionnaire adapted from the Basic Screening Survey. | parents of third- and eighth-grade children in Jeddah (n = 1,668) |
| 3 | Vieira, J. M. R. | Brazil | examine the association of contextual and individual determinants with non-utilization of dental services | Cross-sectional study | adults aged 35-44 years (N = 7,265) from the 2010 Brazilian Oral Health Survey |
| 4 | Rostam Beigi, M. | Iran | Investigate the relationship between complementary health insurance and frequency of dental visits |  |  |
| 5 | Rezaei, S. | Iran | provide insight into socioeconomic inequalities in dental care utilization and its main contributing factors among Iranian households. | Cross-sectional study | A total of 37,860 households from the 2017 Household Income and Expenditure Survey |
| 6 | Nyamuryekung'e, K. K. | Tanzania | determine the direct and indirect costs of accessing and utilizing dental services | Cross-sectional study | 489 dental patients utilizing an out-of-pocket payment modality was carried out in four regional hospitals |
| 7 | Mulyanto, J. | Indonesia | assess socioeconomic inequalities in the utilization of primary care, secondary care and preventive care | Cross-sectional study | data from the 2014 Indonesia Family Life Survey with a total of 42 083 adult participants. |
| 8 | Motlagh, S. N. | Iran | achieve evidence-based policymaking to improve access to dental services and reduce inequality in the utilization of these services | Cross-sectional study | 500 households in Khorramabad city in 2017 |
| 9 | Medina-Solís, C. E. | Mexico | estimate the Out-Of-Pocket Expenditures (OOPEs) incurred by households on dental care | Cross-sectional study | 763 schoolchildren |
| 10 | Mardian, A. | Indonesia | evaluated factors contributing to oral health service utilization for elderly in elderly | Cross-sectional study | data for 291 elderly individuals was obtained by screening the dental and oral health statuses, |
| 11 | Maffioletti, F. | Brazil | test the relationship of predisposing, enabling and need characteristics with dental services utilization | Cross-sectional study | 358 children enrolled in public schools located in a deprived area |
| 12 | Klint, N. K. | Tanzania | To determine the direct and indirect costs of accessing and utilizing dental services | Cross-sectional study | Survey of 489 dental patients utilizing an out-of-pocket payment modality was carried out in four regional hospitals |
| 13 | Gallego-Gómez, C. L. | Colombia | Determining processes of access to dentistry in boys and girls during early childhood | Mixed method |  |
| 14 | Comassetto, M. O. | Brazil | assess the access and factors connected to dental visits in children | Cross-sectional study | 10 Basic Health Units through a questionnaire and clinical examination on 560 children. Clinical |
| 15 | Amornsuradech, S. | Thailand | determine the relationship between socioeconomic status (SES) and oral health | Cross-sectional | secondary data from the 7th Thailand National Oral Health Survey (2012). |
| 16 | Xu, M. R. | China | explore the patterns of oral health service utilization and to determine the related factors among preschool | Cross-sectional | 1425 preschool children aged 2 to 6 years was carried out in five kindergartens |
| 17 | Teixeira, A. K. M. | Brazil | investigate dental care from the life course perspective and its determinant factors among young | Cohort study | 482 young people aged between 17 and 21 years |
| 18 | McMahon, A. D. | Scotland | Inequalities in the dental health needs and access to dental services among looked after children | Cross-sectional | 633 204 children in publicly funded schools in Scotland during the academic year 2011/2012 |
| 19 | Kailembo, A. | China, Ghana and India. | describes socioeconomic inequality in self-reported unmet need for oral health services in adults aged | Cross-sectional | national survey data from the WHO SAGE Wave 1 (2007-2010) was conducted. |
| 20 | Herkrath, F. J. | Brazil | evaluate the association of contextual and individual factors with the utilization of dental services | Cross-sectional | Individual-level data from 27,017 adults residents in the State capitals who were interviewed in the 2013 Brazilian National Health Survey |
| 21 | Cheng, M. L. | China | evaluate the use of oral health services, the economic burden of oral diseases and related influential factors | Cross-sectional | the 4th National Oral Health Survey of China conducted in 2015 to 2016 |
| 22 | Amiresmaili, M. | Iran | investigate the effective factors on utilization of dental services. | Cross-sectional | 1185 household heads were selected randomly using a researcher-made questionnaire based on WHO |
| 23 | Piovesan, C. | Brazil | effect of contextual factors on dental care utilization was evaluated after adjustment for individual characteristics | cross-sectional | 639 preschool children aged 1 to 5 years |
| 24 | de Andrade, F. B. | Brazil | assess socioeconomic inequality in the use of dental care among older Brazilian adults and to analyses the extent to which certain determinants contribute to that inequality | cross-sectional | data from the National Oral Health Survey conducted in 2010 |
| 25 | Rezaei s | Iran | examine the utilization of dental care and identify the main socioeconomic factors affecting the use of these services | cross-sectional survey | multistage sampling was conducted to obtain information on the dental care visits of 520 head of households |
| 26 | Rad, E. H. | Iran | describe inequality in dental care utilization | cross-sectional survey | Data of a health utilization survey which previously had been gathered in Shiraz, Iran were used for this study |
| 27 | Onyejaka, N. K. | Nigeria | identify factors that facilitated and served as barriers to children's utilization of oral health care services | cross-sectional survey | recruited 1406 primary school pupils aged 8 to 11 years |
| 28 | Guan, Y. | China | explore socioeconomic inequalities in dental caries among 5-year-olds in four Chinese | cross-sectional survey | data from 1,732 children living in Guangxi, Hubei, Jilin and Shanxi who participated in the Third National Oral Health Survey in 2005 |
| 29 |  | Iran | identify the socioeconomic distribution of perceived oral health among adults | cross-sectional | elf-report data were obtained from the 2010 dental telephone interview survey. |
| 30 | Molete, M. P. | South Africa | The study sought to determine barriers to accessing oral health services amongst the elderly residing | cross-sectional | Data were collected from questionnaires and clinical oral examinations assessing the DMFT and CPITN scores. |
| 31 | Somkotra, T. | Thailand | o assess the socioeconomic-related inequality in dental care utilization | cross-sectional | data were taken from the nationally representative Thailand Health &amp; Welfare Survey 2007. |
| 32 | Ayo-Yusuf, I. J. | South Africa | determine the contributions of socio-economic position and health insurance enrollment in explaining racial disparities in preventive dental visits | secondary cross-sectional data | Data on the dentate adult population participating in the last South African Demographic and Health Survey conducted during 2003-2004 (n = 6,312) was used |
| 33 | Palmier, A. C. | Brazil | evaluate the association between the proportion of tooth extractions, socioeconomic indicators, and the availability of oral health services in an underprivileged area | ecological study | carried out in 52 municipalities in the state of Minas Gerais, Brazil |
| 34 | Celeste, R. K. | Brazil | evaluate the association between income inequality at a lagged time of 2 and 11 years with two short latency outcomes | cross-sectional | data from the Brazilian oral health survey in 2002-2003. Our analysis included 13,405 subjects aged 35-44 years |
| 35 | Baldani, M. H. | Brazil | assess the role of the individual determinants on the inequalities of dental services utilization among low-income children |  |  |
| 36 | Manoelito Ferreira SILVA(2015) | Brazil | evaluate the association  between social inequality indicators and oral health conditions | Prospective Cohort Study | 248 adults aged between 20–64 years |
| 37 | Luana Leal Roberto | Brazil | analyze tooth loss among Brazilian adults (35–44 years of age), in accordance with individual and contextual | cross-sectional | Brazil |
| 38 | Rossi, T. R. A. | Brazil | analyzed the effects of austerity and economic crisis on the financing of oral health, provision and use of public services and access to exclusively dental plans in Brazil | A retrospective, descriptive study was carried out, with a quantitative approach. | Data were collected from the National Health Funding database, the National Supplementary Health Agency |
| 39 | Lalitha, N. D. | china | The aim of this study was to evaluate the accessibility and barriers to oral health and to evaluate the oral health status of the [Narikurava](https://en.wikipedia.org/wiki/Narikurava)  population | cross-sectional | conducted among gypsy [Narikurava](https://en.wikipedia.org/wiki/Narikurava)  population in Chennai. The sample size was 102 |
| 40 | Gallego-Gómez, C. L. | Colombia | understand structural, specific and singular processes that determine access to dental care in early childhood | Mix method | group interviews and interviews with coordinators and parents were conducted. |
| 41 | Comassetto, M. O. | Brazil | assess the access and factors connected to dental visits in children up to age five | cross-sectional study | The cross-sectional study was conducted in 10 Basic Health Units through a questionnaire and clinical examination on 560 children |
| 42 | Gao, X. | china | evaluate dental utilization among 3-,4-, and 5-year-old children in China and to use Andersen's behavioral model to explore influencing factors, | cross-sectional study | Data of 40,305 children aged 3-5 years were extracted from the Fourth National Oral Health Survey, |
| 43 | Dho, M. S. | Argentina | analyze the factors associated with the use of dental health services (UDHS) by adults | cross-sectional study | information concerning the study variables was collected via a home survey. The sample size was established with a 95% confidence interval level |
| 44 | Rebelo Vieira, J. M. | Brazil | examine the association of contextual and individual determinants with non-utilization of dental services | cross-sectional study | Data were from adults aged 35-44 years (N = 7,265) from the 2010 Brazilian Oral Health Survey (SB Brazil Project). |
| 45 | Li, C. F. | china | investigate inequality in dental service utilization in Chinese middle-aged and senior adults and changes in inequality over time and to determine the sources of inequality | cross-sectional study | data included 17 648 individuals aged 45 years and older in 2013 and 15 450 individuals in 2015 who participated |
| 46 | Leinsalu, M. | Lithuania | assess trends and inequalities in dental care utilization | cross-sectional | Data on 22,784 individuals in the 20-64 age group were retrieved from nationally |
| 47 | Janakiram, C. | India | examine the political priority of oral health in India and to understand the underlying reasons for the political support oral health receives | analysis is based on the political power framework |  |
| 48 | Feldens, C. A. | Brazil | Identify contextual, socioeconomic, and child characteristics associated with dental visitation | cross-sectional | Within a Brazilian birth cohort (N = 435), multivariable regression models |
| 49 | Babu, N. K. | china | assess the relationship between oral health-related quality of life (OHRQoL) and oral health status | cross-sectional study | total population census and list of houses in Poonamallee were obtained from the respective authorities. |
| 50 | Ahmad, M. S. | Malaysia | review discusses government policies and services available to support oral health needs of the elderly population | review |  |
| 51 | Shetty, N. | India | evaluate an association between the level of education (educational qualification) and utilization of restorative dental care among rural women | cross-sectional survey | A semi-structured questionnaire was administrated to 660 rural women associated with self-help group by trained research assistants |
| 52 | Marino, R. | Chile | factors associated with their use of oral health care services, and self-reported barriers to using oral health care services. | cross-sectional survey | 438 older adults, aged 65-74 years, living independently in the community were orally examined and underwent an oral health interview. |
| 53 | Bhatt, S. | India | assess factors influencing the oral health and utilization patterns of oral health services | A house-to-house survey | 840 individuals in fishermen population. Oral health status was evaluated |
| 54 | Ali, H. M. | Sudan | describe the patterns of oral-health-related background factors in children with and without CHD and explore any differences, and to evaluate the effects of background factors | cross-sectional study |  |
| 55 | E.O. Ogunbodede1 | Nigeria | Oral Health Inequalities between  Rural and Urban Populations of the  African and Middle East Region | literature review |  |
| 56 | Granville-Garcia, A. F. | Brazil | evaluate the influence of oral problems and biopsychosocial factors on the use of dental services by preschoolers | cross-sectional | carried out with preschoolers in northeastern Brazil. Parents answered questions on visits to the dentist and sociodemographic characteristics, and completed the Brazilian version of the Early Childhood Oral Health Impact Scale |
| 57 | Cornejo-Ovalle, M. | Chile | examines changes in the distribution and socioeconomic inequalities of dental care utilization among adults | cross-sectional |  |
| 58 | Rocha-Buelvas, A. | Colombia | To analyze determinants of access to oral health care among university students in municipality of Pasto. |  | sample of 338 university students answered a confidential survey that was based upon previous studies using a health care services utilization |
| 59 | Qiu, R. M. | china | Factors related to children's caries: A structural equation modeling approach | cross-sectional | A model which explored the factors related to children's dental caries was tested in this study usin structural equation modeling. |
| 60 | Somkotra, T. | Thai | determine factors associated with the observed inequality after the country achieved universal coverage. | cross-sectional | Data of 10096 Thai elderly (aged over 60years) were selected. Descriptive analyses of the features of dental care utilization among Thai elderly were carried out, |
| 61 | Ola, D. | Nigeria | test the relationship between socioeconomic position (SEP), family composition, number of siblings, and birth position in the family, and the utilization of oral health | cross-sectional study | study design included senior secondary school pupils in the Central Local Government Area |
| 62 | Peres, K. G. | Brazil | analyze access to and utilization of dental care services | cross-sectional study | used data from the 2003 and 2008 Brazilian National Household Surveys, which we compared to data from the 1998 survey |
| 63 | Piovesan, C. | Brazil | The influence of socioeconomic factors and self-rated oral health on children's dental health assistance was assessed. | cross-sectional | Data about the use of dental service, socioeconomic status, and self-perceived oral health were collected by means of structured interviews |
| 64 | Maharani, D. A. | Indonesia | provide evidence of socioeconomic-related inequality and horizontal inequity in dental care utilization among Indonesian adults who reported having had dental problems | cross-sectional | data from the Indonesian National Socio-Economic Survey 2004 and from the Indonesian Medical Council. Respondents included individuals at least 15 years old |
| 65 | Baldani, M. H. | Brazil | To assess the association between socioeconomic indicators of provision of public dental services and allocation of financial resources in health | cross-sectional ecological study | data obtained from the Brazilian Ministry of Health data system for 399 cities in the State of Paraná (Southern Brazil) between 1998 and 2005 |
| 66 | Adeyemi, A. T. | Nigeria | influence of socio-economic status on the utilization of orthodontic services and the uptake of orthodontic treatment | cross-sectional |  |
| 67 | Tsai, W. C. | Taiwan | investigated the influence of medical market competition on the utilization of dental care | cross-sectional | Study used the healthcare sub-regions (HCSRs) in the healthcare net as the observation units |
| 68 | Souza, J. G. S. | brazil | describe the use of dental care service during early childhood and the possible socioeconomic inequalities in this use. | cross-sectional | Data from the Brazilian Oral Health Survey (SB Brazil, 2010) were used. Interviews with the children's parents and clinical examinations with the children were conducted in a 5-year-old representative sample. |
| 69 | Machry, R. V. | brazil | assessed the relationships between socioeconomic and psychosocial factors and the utilization of dental health services | cross-sectional | Data were collected through clinical exams and a structured questionnaire administered during the National Day of Children's Vaccination. |
| 70 | Roncalli, A. G | Brazil | evaluate the social determinants of dental treatment needs in 35-44 year old Brazilian adults, assessing whether inequalities in needs are expressed at individual and contextual levels. | cross-sectional |  |
| 71 | Villalobos-Rodelo, | Mexico | identify the effect of unmet dental treatment needs and socioeconomic and sociodemographic variables on the patterns of dental visits | case-control study | included 379 patients that had a dental visit because of dental pain in the 12 months preceding this study and 1,137 controls |
| 72 | Ferreira, C. O. | Brazil | To assess the association between recent use of dental services, socioeconomic factors and oral health measures among elderly | cross-sectional | The sample consisted of elderly individuals aged 65 to 74, who participated in the Brazilian Oral Health Survey in 2010 |
| 73 | Medina-Solís, C. E. | Mexico | identify preventive and curative dental health service utilisation (DHSU) in the context of associated clinical and non-clinical factors among adolescents and young adults | cross-sectional | Participants were 638 adolescents and young adults aged 16-25 randomly selected from university applicants. |
